# Postoperative adjuvant therapy for stage IA-IIA cervical adenocarcinoma (FIGO 2018) with one intermediate-risk factor: a multicentre retrospective cohort study of 63,926 cases

**DOI:** 10.1186/s12957-025-03739-9

**Published:** 2025-03-14

**Authors:** Jiaxin Fu, Cong Liang, Lixin Sun, Hongwei Zhao, Zhumei Cui, Jinghe Lang, Chunlin Chen, Ping Liu

**Affiliations:** 1 Department of Obstetrics and Gynecology, Nanfang Hospital, Southern Medical University, No. 1838, Guangzhou Avenue, Guangzhou, Guangdong 510515 China; 2 Department of Gynecologic Oncology, Shanxi Provincial Cancer Hospital, Taiyuan, China; 3 Department of The Affiliated Hospital Of Qingdao University, Shandong, China; 4 Department of Obstetrics and Gynecology, Peking Union Medical College Hospital, Beijing, China

**Keywords:** Cervical cancer, Adenocarcinoma, FIGO 2018, Intermediate risk factors, Adjuvant therapy

## Abstract

**Objective:**

To compare the 5-year oncological outcomes of different adjuvant treatment modalities in patients with FIGO 2018 stage IA-IIA cervical adenocarcinoma who underwent open radical hysterectomy and one intermediate-risk pathological factor.

**Methods:**

Based on the Four C database (between 2004 and 2018,*n*=63,926), patients with FIGO 2018 stage IA-IIA cervical adenocarcinoma and only one intermediate-risk pathological factor underwent open extensive hysterectomy. All patients were divided into three groups, namely, the simple surgery group (radical hysterectomy, RH), postoperative adjuvant chemotherapy group (radical hysterectomy and chemotherapy, RH + CT), and postoperative adjuvant chemoradiotherapy group (radical hysterectomy and radiotherapy/concurrent chemoradiotherapy, RH + RT/CCRT). The 5-year OS and DFS rates were compared among the three groups.

**Results:**

Of the 219 cervical adenocarcinoma patients with only one intermediate-risk pathological factor, 50 patients had RH; 54 patients had RH + CT; and 115 patients had RH + RT/CCRT. There were no significant differences in 5-year OS and 5-year DFS rates among the three groups (RH vs. RH + CT: 92.7% vs. 90.3%, *P* = 0.749; 88.5% vs. 85.1%, *P* = 0.680, RH vs. RH + RT/CCRT: 90.7% vs. 82.3%, *P* = 0.484; 84.4% vs. 90.1%, *P* = 0.494, RH + CT vs. RH + RT/CCRT: 89.9% vs. 90.6%, *P* = 0.815; 90.5% vs. 90.8%, *P* = 0.905).

**Conclusion:**

Postoperative adjuvant chemotherapy or chemoradiotherapy did not significantly improve the outcomes of FIGO 2018 IA-IIA cervical adenocarcinoma patients with only one intermediate risk factor.

## Introduction

Cervical adenocarcinoma (AC) is a major pathological subtype of cervical cancer, second only to squamous cell carcinoma (SCC). In recent years, its incidence has been steadily increasing, accounting for approximately 10–25% [1]. According to the 2025 National Comprehensive Cancer Network (NCCN) guidelines, the recommended postoperative adjuvant therapy for AC is consistent with SCC. For early-stage AC patients, adjuvant therapy is recommended if one high-risk pathological factor or two or more intermediate-risk pathological factors are present postoperatively. This treatment involves postoperative pelvic external beam radiation therapy, with or without concurrent platinum-based chemotherapy [2].

However, current clinical practice faces two significant challenges. First, most research has primarily focused on patients with two or more intermediate-risk factors, leaving the optimal management strategy for those with a single intermediate-risk factor unclear. Moreover, regional guidelines offer conflicting recommendations. For instance, the European Society for Medical Oncology (ESMO) suggests that patients with a single intermediate-risk factor do not require postoperative adjuvant treatment. In contrast [3], the Japanese Society of Gynecologic Oncology (JSGO) guidelines recommend adjuvant treatment for any patient with at least one intermediate-risk factor [4], while the International Federation of Gynecology and Obstetrics (FIGO) advises radiotherapy for patients with any two intermediate-risk factors [5]. Second, AC and SCC exhibit distinct biological behaviors [6, 7]. The overall survival rate for AC at each stage is 10–20% lower than that for SCC [8, 9], likely due to AC’s higher risk of lymph node metastasis [10]. Notably, some guidelines classify AC itself as an independent intermediate-risk factor, which may alter the decision threshold for adjuvant therapy when combined with other intermediate-risk indicators. However, existing studies often mix different pathological types, overlooking the unique characteristics of adenocarcinoma. Some scholars have demonstrated that AC is less sensitive to radiotherapy alone compared to SCC [11, 12], though other studies suggest that postoperative radiotherapy can improve prognosis. As a result, there is no consensus on whether AC patients with a single intermediate-risk factor require adjuvant therapy [13, 14].

To address these issues, this study analyzed cervical adenocarcinoma cases (FIGO 2018 stages IA-IIA) from 47 hospitals in mainland China. By comparing 5-year survival outcomes of radical hysterectomy (RH), RH with chemotherapy (CT), and RH with radiotherapy or chemoradiotherapy (RT/CCRT), it aims to fill the evidence gap in postoperative treatment and provide more precise, evidence-based strategies to improve patient outcomes.

## Materials and methods

### Data collection

The Four C Database is a multicenter retrospective cohort study, launched on March 8, 2015, approved by the Ethics Committee of Southern Hospital of Southern Medical University, ethics number NEEC-2017-135, clinical trial registration number CHiCTR1800017778 (Inter-national Clinical Trials Registry Platform Search Port, http://apps.who.int/trialsearch/). Case data collection methods refer to the published articles of our team [15, 16].

### The patient and public involvement section

We did not involve patients or the public in our work.

### Inclusion criteria and exclusion criteria

Inclusion Criteria were as follows: age ≥ 18 years old, cervical adenocarcinoma, FIGO 2018 stage IA1 (LVSI+)-IIA2 stage, no preoperative adjuvant therapy, abdominal surgery, QM-B or QM-C hysterectomy and pelvic lymphadenectomy with or without para-aortic lymphadenectomy/biopsy, the postoperative pathological report was complete and the lymph node status was negative, high-risk factors such as parametrial metastasis and vaginal metastasis are negative, and there is only one intermediate-risk factor (LVSI(+),deep stromal invasion or tumor size > 4 cm). Exclusion criteria were as follows: did not meet the inclusion criteria; pregnancy with cervical cancer, cervical stump cancer, or combined with other malignant tumors.

Three groups of patients were included in the study: RH group: no adjuvant therapy after surgery; RH + CT group: only adjuvant chemotherapy (≥ 2 courses), no other adjuvant therapy; RH + RT/CCRT: Only adjuvant radiotherapy/chemoradiotherapy, no other adjuvant therapy.

### Propensity score matching

In order to reduce the influence of baseline differences, after the real-world study, the variable factors of age, uterine type, and FIGO stage were included into the logistic regression model for 1:1 propensity score matching [17]. Caliper matching was used, and the caliper value was 0.05.

### Statistical analysis

Statistical analysis was performed using SPSS software (version 23.0, USA). Continuous variables were expressed as mean ± standard deviation, and independent samples t-test was used for comparison between groups; categorical variables was expressed as percentage (%), and chi-square test or Fisher’s exact test was used for comparison of rates between groups. The median follow-up times were expressed by the reverse Kaplan-Meier method; survival analysis was performed by Kaplan-Meier method; the hazard ratio (HR) and 95% CI were calculated by multivariate Cox regression model to identify independent influencing factors. *P* < 0.05 was considered statistically significant.

## Results

### Study population

**Fig. 1:**
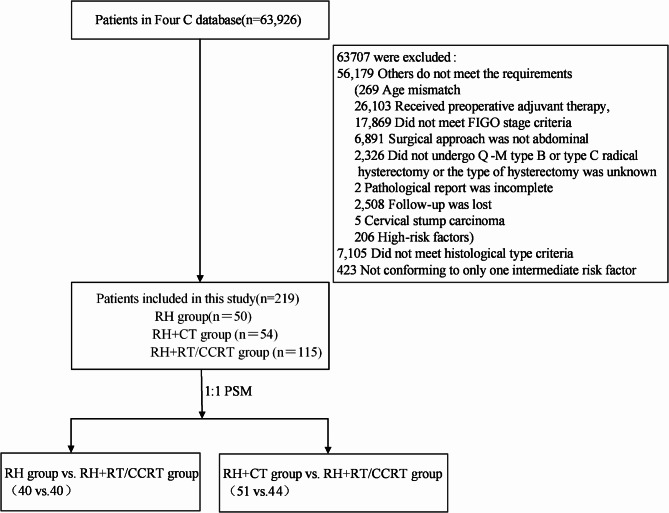
Flow diagram of recruitment and exclusions

Among the 63,926 patients from 47 hospitals were in the dataset, adenocarcinoma (*n*=642,8.3%), squamous cell carcinoma (n=6943 cases,89.5%) and adeno-squamous cell carcinoma (n=162,2.2%) were preliminarily screened. Further screening revealed that 219 of the 642 adenocarcinoma patients had only one medium risk factor (including 177 with positive cervical invasion, 12 with positive LVSI, and 30 with tumor diameter > 4 cm). According to the postoperative treatment, the patients were divided into three groups:50 cases in the RH group, 54 cases in the RH + CT group, and 115 cases in the RH + RT/CCRT group (Fig. 1).

### Comparison of oncological outcomes between the RH and RH + CT groups

Baseline analysis between the RH group (*n*=50) and the RH + CT group (n=54)showed that there was no significant difference in age, FIGO stage, and type of surgery (*P* > 0.05). Propensity score matching (PSM) (1:1) was not performed because all baseline characteristics were balanced (Table 1).

**Table 1:** Comparison of the clinical characteristics and pathological outcomes between RH group, RH+CT group and RH+RT/CCRT group

	Before matching				Before matching				After Matching				Before matching				After Matching			
	RH (*n*=50,%)	RH+CT (*n*=54,%)	*P*		RH (*n*=50,%)	RH+RT/CCRT (*n*=115,%)	*P*		RH (*n*=40,%)	RH+RT/CCRT (*n*=40,%)	*P*		RH+CT (*n*=54,%)	RH+RT/CCRT (*n*=115,%)	*P*		RH+CT (*n*=51,%)	RH+RT/CCRT (*n*=44,%)	*P*	
**Age** **(years)**	48.00±8.95	47.57±9.60	0.816		48.00±8.95	47.49±9.35	0.743		47.23±9.37	47.78±8.63	0.786		47.57±9.60	47.49±9.35	0.955		46.63±9.01	49.98±9.30	0.078	
**FIGO stage**			0.330				**0.000**				1.000				**0.002**				0.831	
IA2	1(2.0)	0(0.0)			1(2.0)	0(0.0)			-	-			-	-			-	-		
IB1	10(20.0)	12(22.2)			10(20.0)	22(19.1)			10(25.0)	10(25.0)			12(22.2)	22(19.1)			12(23.5)	12(27.3)		
IB2	20(40.0)	20(37.0)			20(40.0)	67(58.3)			19(47.5)	19(47.5)			20(37.0)	67(58.3)			20(39.2)	17(38.6)		
IB3	13(26.0)	9(16.7)			13(26.0)	5(4.3)			5(12.5)	5(12.5)			9(16.7)	5(4.3)			9(17.6)	5(11.4)		
IIA1	6(12.0)	10(18.5)			6 (12.0)	21(18.3)			6(15.0)	6 (15.0)			10(18.5)	21(18.3)			10 (19.6)	10 (22.7)		
IIA2	0(0.0)	3(5.6)			-	-			-	-			3(5.6)	0(0.0)			-	-		
**Hysterectomy**			0.475				**0.001**				1.000				**0.01**				0.721	
QM-B	29(58.0)	35(64.8)			29(58.0)	95(82.6)			25(62.5)	25(62.5)			35(64.8)	95(82.6)			33(64.7)	30(68.2)		
QM-C	21(41.0)	19(35.2)			21(42.0)	20(17.4)			15(37.5)	15(37.5)			19(35.2)	20(17.4)			18(35.3)	14(31.8)		

RH, Radical Hysterectomy; RH+CT, Radical Hysterectomy and chemotherapy; RH+RT/CCRT, Radical Hysterectomy and radiotherapy /concurrent chemoradiotherapy

**Fig. 2:**
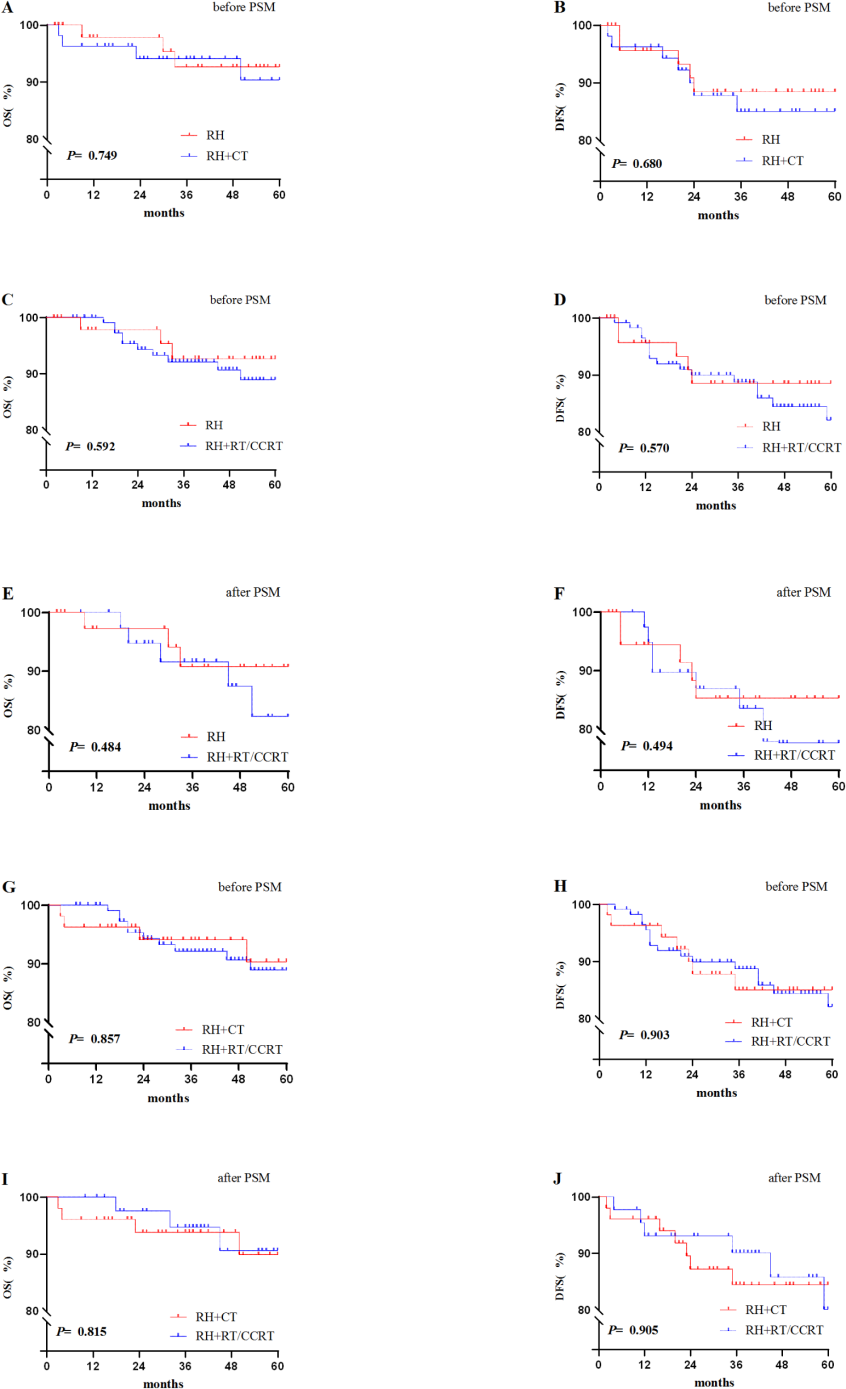
Before and after PSM, the Kaplan-Meier survival curves of the three groups

The median follow-up times were 52 months (RH vs. RH + CT: 55months vs. 49 months). There was no significant difference in 5-year OS and 5-year DFS between the two groups (92.7% VS. 90.3%, *P* = 0.749, Figs. 2A and 88.5% VS. 85.1%, *P* = 0.680, Fig. 2B).

### Comparison of oncological outcomes between the RH and RH + RT/CCRT groups

The median follow-up times were 52 months (RH vs. RH + RT/CCRT: 55 months vs. 50 months). There was a significant difference between the RH group (*n*=50) and the RH + RT/CCRT group (n=115) in terms of FIGO stage and type of surgery, and these differences were balanced after 1:1 PSM (Table 1). The Kaplan-Meier curves showed that there was no significant difference in the 5-year OS and 5-year DFS(92.7% VS. 88.9%, *P* = 0.592, Figs. 2C and 88.5% VS. 82.1%, *P* = 0.570, Fig. 2D)。.

After 1:1 matching, 40 patients were in the RH group, 40 patients were in the RH + RT/CCRT group, and there was no significant difference in 5-year OS (90.7% VS. 82.3%, *P* = 0.484, Fig. 2E) or 5-year DFS (84.4% VS. 90.1%, *P* = 0.494, Fig. 2F) between the two groups.

### Comparison of oncological outcomes between the RH + CT and RH + RT/CCRT groups

The median follow-up times were 50 months (RH + CT vs. RH + RT/CCRT: 49 months vs. 50 months). There was no significant difference in the 5-year OS and 5-year DFS(90.3% VS 88.9%,*P* = 0.857, Figs. 2G and 85.1% VS 82.1%, *P* = 0.903, Fig. 2H).

Next, 1:1 PSM was used to reduce age bias. After matching, 51 patients were included in the RH + CT group and 44 patients in the RH + RT/CCRT group, and the clinicopathologic variables were well balanced between the two groups (Table 1). There was no significant difference in 5-year OS (89.9% VS 90.6%, *P* = 0.815, Fig. 2I) or DFS rate (90.5% VS 90.8%, *P* = 0.905, Fig. 2J) between the two groups.

### Multivariate analysis between three groups

In the Cox multivariate analysis, postoperative adjuvant chemoradiotherapy or radiotherapy /concurrent chemoradiotherapy was not associated with 5-year OS and 5-year DFS in both the unadjusted and adjusted analyses (*P*>0.05) (Table 2).

**Table 2:** Multivariate analyses of outcomes between RH group, RH+CT group and RH+RT/CCRT group

		Before matching						After Matching					
		OS			DFS			OS			DFS		
		OR	95%CI	P	HR	95%CI	P	OR	95%CI	P	HR	95%CI	P
RH vs. RH+CT	age	1.021	0.935∼1.115	0.641	1.029	0.964∼1.098	0.393						
	hysterectomy types	0.705	0.134∼3.722	0.681	1.955	0.615∼6.215	0.256	—					
	treatment	1.390	0.297∼6.516	0.676	1.140	0.337∼3.861	0.833						
RH vs. RH+RT/CCRT	age	1.058	0.998∼1.112	0.057	1.029	0.982∼1.077	0.240	1.043	0.568∼1.138	0.341	1.037	0.972∼1.106	0.272
	ysterectomy types	0.601	0.129∼2.803	0.517	1.244	0.473∼3.274	0.658	0.424	0.084∼2.135	0.298	0.703	0.220∼2.245	0.552
	treatment	0.585	0.151∼2.274	0.439	0.715	0.250∼2.048	0.532	0.991	0.204∼4.827	0.991	1.076	0.321∼3.606	0.905
RH+CT vs. RH+RT/CCRT	age	1.076	1.019∼1.136	0.009	1.048	1.004∼1.094	0.033	1.077	0.994∼1.166	0.069	1.060	1.000∼1.125	0.052
	hysterec tomytypes	1.309	0.402∼4.263	0.655	1.730	0.727∼4.118	0.216	1.523	0.299∼7.752	0.612	2.645	0.840∼8.333	0.097
	treatment	1.059	0.318∼3.520	0.926	1.024	0.411∼2.554	0.959	0.677	0.149∼3.082	0.614	0.692	0.219∼2.185	0.531

RH: Radical Hysterectomy; RH+CT: Radical Hysterectomy and chemotherapy; RH+RT/CCRT: Radical Hysterectomy and radiotherapy /concurrent

## Discussion

The study retrospectively analyzed the clinical data of 219 patients with FIGO 2018 IA-IIA cervical adenocarcinoma from 2004 to 2018. In a real-world study, the three groups of RH, RH + CT, and RH + RT/CCRT were compared. We found that there was no difference in the 5-year OS and 5-year DFS rates between the three groups, and Cox multivariate analysis showed that the postoperative adjuvant chemoradiotherapy or radiotherapy/concurrent chemoradiotherapy was not associated with 5-year oncological outcomes. Therefore, this study revealed that postoperative adjuvant chemotherapy or chemoradiotherapy did not significantly improve the outcome of FIGO 2018 IA-IIA cervical adenocarcinoma patients with only one intermediate risk factor.

The NCCN guidelines recommended that the postoperative adjuvant treatment plan for cervical adenocarcinoma is the same as that for cervical squamous cell carcinoma [2]. If there is one high-risk pathological factor or two or more intermediate-risk pathological factors after surgical treatment of early cervical adenocarcinoma, the supplemental treatment of the pelvic cavity should be performed after surgery, such as external beam ± platinum-containing concurrent chemotherapy (evidence level 2B for concurrent chemotherapy). However, there is no recommendation for postoperative adjuvant therapy for only one intermediate-risk pathological factor.

Adenocarcinoma itself may be an intermediate-risk factor; when adenocarcinoma is combined with any other intermediate-risk factors, it will affect the decisions of adjuvant therapy. However, the guidelines do not mention whether postoperative adjuvant therapy should be administered to patients with cervical adenocarcinoma with only one intermediate-risk pathological factor, and no relevant studies have been reported in the literature. Therefore, it is necessary to discuss the treatment measures for these cases.

Radical hysterectomy is the first choice for the treatment of early cervical adenocarcinoma; the curative effect of surgery for locally advanced adenocarcinoma is also better than that of radiotherapy alone [18].In our study, in cervical adenocarcinoma patients with only one intermediate-risk factor, the effect of surgery alone was comparable to that of postoperative adjuvant chemotherapy. Zhou’s study [19] also confirmed that for cervical adenocarcinoma, regardless of tumor diameter, there was no significant difference in survival between patients who received RH + RT and those who received RH alone. Surgery is the best treatment for local early-stage cervical adenocarcinoma. However, the study did not further analyze the patients with postoperative pathologically suggested intermediate risk factors. The selection of appropriate adjuvant therapy according to postoperative risk factors is very important for the prognosis of patients. Nasioudis [20] selected 765 cases of FIGO 2018 stage IB cervical cancer with one or more intermediate risk factors, including 186 cases (24.3%) of adenocarcinoma, and found that postoperative adjuvant radiotherapy or chemoradiotherapy did not improve the outcome did not significantly improve the outcome (4-year OS:87.1% vs. 88.4%, *P* = 0.44). Moreover, the research found that for patients who received adjuvant radiotherapy, there was no difference between those who received concurrent chemotherapy and those who did not (4-year OS: 89.8% vs. 86.3%, *P* = 0.36). Our results also support the opinion, with comparable oncological outcomes observed for the RH group, RH + CT group, and the RH + RT/CCRT group. Additionally, we only included adenocarcinoma cases in this study to avoid other possible confounding factors.

Moreover, we found that for cervical adenocarcinoma with an intermediate risk factor, postoperative chemoradiotherapy and chemotherapy had comparable oncologic outcomes (OS: 89.9% vs. 90.6%, *P* = 0.815; DFS: 90.5% vs. 90.8%, *P* = 0.905). Similar to our results, Koji Matsuo [21] selected 555 cases of cervical cancer stage IB with intermediate risk factors, including 133 cases (24.7%) of adenocarcinoma. All patients received chemotherapy, chemoradiotherapy, and radiotherapy alone after RH, and the results showed that the OS rate of chemotherapy group was similar to that of the concurrent chemoradiotherapy group (5-year OS: 88.1% vs. 90.2%, HR 0.98, 95%CI 0.52–1.83, *P* = 0.94) as well as the disease-specific survival (95.4% vs. 94.8%, HR 0.85, 95%CI 0.34–2.07, *P* = 0.71). However, this study did not limit the number of intermediate risk factors for a more detailed analysis. Another study [22] by retrospectively analyzed 571 cases of stage I-IIA cervical cancer with intermediate risk factors, including adenocarcinoma cases (59, 10.3%), and found that the prognosis of patients who received adjuvant therapy after surgery was better than those who did not receive adjuvant therapy. For adjuvant therapy, the efficacy of chemotherapy/chemotherapy combined with radiotherapy was better than radiotherapy alone (5-year PFS: 90.4%, 5-year OS: 90.9%). Ryu [13] also asserted that postoperative radiotherapy and chemotherapy can improve the prognosis of patients. This is inconsistent with our study, which may be because these studies included other pathological types such as squamous cell carcinoma, adeno-squamous cell carcinoma, etc., but did not explore the special pathological type of adenocarcinoma alone. The biological characteristics of adenocarcinoma are different from those of squamous cell carcinoma, and it may also be an intermediate-risk factor itself, so these factors may lead biased the results. Additionally, these studies are mostly based on the old FIGO staging, which may not be applicable to patients classified with the FIGO 2018 staging system.

We acknowledge that there are several limitations to this study. First, this is a retrospective study, so there may be confounding factors and biases. Second, this study does not fully cover all regions in China, but the database covers 47 hospitals in China, which is representative of the diagnosis and treatment of cervical cancer patients across China. This retrospective study was based on a large sample from multiple centers and carried out strict PSM, which not only reduced the bias that can arise due to small sample sizes and single-center data but also better balanced confounding factors such as baseline differences.

Recent studies have confirmed that Pembrolizumab combined with platinum-based chemotherapy, with or without Bevacizumab, can serve as a first-line treatment for persistent, advanced, metastatic, or recurrent cervical cancer with PD-L1 positivity (CPS ≥ 1) [23]. Additionally, multiple studies have shown that immunotherapy can enhance the efficacy of chemoradiotherapy, and bevacizumab also demonstrates significant prognostic benefits [24, 25]. Further exploration is warranted to evaluate the effects of combining immunotherapy with chemoradiotherapy in earlier stages of cervical cancer. We also anticipate more international prospective studies in this field.

## Conclusion

Among patients with FIGO 2018 stage IA-IIA cervical adenocarcinoma with only one intermediate risk factor, postoperative adjuvant chemotherapy or chemoradiotherapy did not significantly improve the outcomes.

## Data Availability

No datasets were generated or analysed during the current study.

## Declarations

### Strengths and limitations of this study

(1) Adjuvant therapy remains unknown in cervical adenocarcinoma patients. (2) Radical hysterectomy alone can be used to treat cervical adenocarcinoma. (3) Postoperative adjuvant treatment did not improve the outcome.

### Data sharing

The datasets used and/or analysed during the current study are available from the corresponding author on reasonable request.

### The patient and public involvement section

We did not involve patients or the public in our work.
